# Use of Anticoagulation for Secondary Stroke Prevention in Cerebral Cavernous Malformations: A Case Report

**DOI:** 10.7759/cureus.78913

**Published:** 2025-02-12

**Authors:** Lisle W Blackbourn, Melissa Kim, Nicholas J Comardelle, Deepak Reddy

**Affiliations:** 1 Neurology, University of Illinois College of Medicine Peoria, Peoria, USA; 2 Neurology, OSF Illinois Neurological Institute, Peoria, USA; 3 Medicine, University of Illinois College of Medicine Peoria, Peoria, USA; 4 Neurosurgery, University of Illinois College of Medicine Peoria, Peoria, USA

**Keywords:** anticoagulation, atrial fibrillation, cerebral cavernous malformation, secondary stroke prevention, stroke

## Abstract

The use of anticoagulation for secondary stroke prevention in patients with cerebral cavernous malformations remains a complex and challenging clinical scenario due to fear of hemorrhagic complications. Independent bleeding risks to take into account include patient age, infratentorial location, cerebral cavernous malformation size, and the association with a developmental venous anomaly. Multidisciplinary consultation involving input from neurology, neurosurgery, cardiology, and radiology is also often crucial to optimize patient outcomes in this setting. Here, we present a case of a 58-year-old woman with known cerebral cavernous malformations presenting with a posterior cerebral artery ischemic stroke secondary to atrial fibrillation to discuss the safety of anticoagulation for secondary stroke prevention in such cases.

## Introduction

Cerebral cavernous malformations (CCMs) are the second most incidentally found vascular findings on MRI brain [1,2]. CCM can cause seizures, hemorrhage, or focal deficits due to mass effect or be asymptomatic [2]. It is believed to have a prevalence of up to 0.8% in the general population, an estimated annual risk for symptomatic hemorrhage of up to 3.2%, and a risk of rupture of up to 6% [1,3]. The first-line treatment in CCM is microresection. Due to this risk of hemorrhage, many clinicians might be hesitant to start anticoagulation in such patients.

Here, we present a case of a 58-year-old woman with known CCM presenting with a posterior cerebral artery (PCA) ischemic stroke secondary to atrial fibrillation to discuss the safety of anticoagulation for secondary stroke prevention in such cases.

## Case presentation

**Figure 1:**
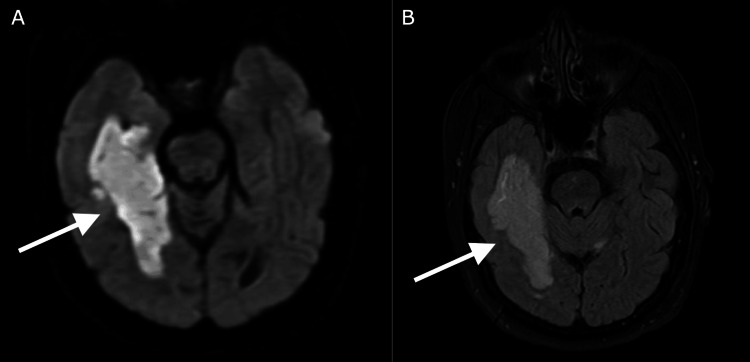
MRI brain with arrows indicating a right posterior cerebral artery territory infarct with (A) DWI hyperintensity and (B) corresponding T2 FLAIR sequencing hyperintensity. DWI: diffusion-weighted imaging; FLAIR: fluid-attenuated inversion recovery

**Figure 2:**
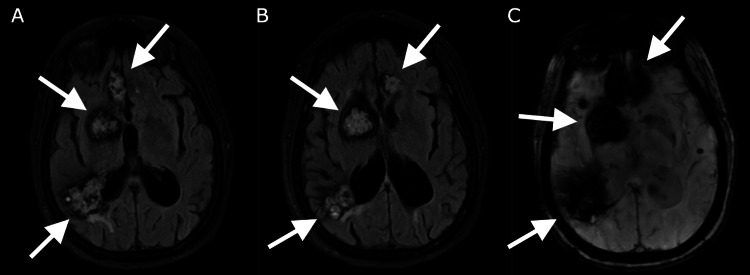
MRI brain with arrows indicating the patient’s known cerebral cavernous malformations as seen on (A and B) T2 FLAIR sequencing and (C) SWAN. FLAIR: fluid-attenuated inversion recovery; SWAN: susceptibility-weighted angiography

The patient is a 58-year-old woman with known CCMs, atrial fibrillation not on anticoagulation, and prior stroke due to atrial fibrillation with residual aphasia who presented to the hospital for acute-onset symptoms of dizziness and left-sided vision loss. On initial assessment, the patient was found to have left homonymous hemianopia. A non-contrast CT head demonstrated no acute abnormalities and no changes to her calcified lesions. CT angiogram of the head and neck demonstrated P1 occlusion of the right PCA. Antithrombotic was not given as the patient unfortunately presented six hours after symptom onset and outside the window and was not a candidate for thrombectomy due to a low National Institutes of Health Stroke Scale (NIHSS) score (NIHSS score of 2 for hemianopia). MRI brain taken the day after symptom onset showed a stable infarct involving the right medial temporal lobe and right inferior occipital lobe as well as a small portion of the left superior cerebellum as seen in Figure 1, and the patient’s known CCMs are seen in Figure 2.

Neurosurgery reviewed the imaging and recommended no active intervention on the patient’s CCMs. In further discussions with the patient, she said she had not been put on anticoagulation for her atrial fibrillation due to her CCM history. It was then found that no literature suggested an increased risk of hemorrhage on anticoagulation/antiplatelets with known cavernomas and that the patient should be on anticoagulation medication due to her atrial fibrillation for secondary stroke prevention. It was also discussed that there likely would be an increased risk of mortality/morbidity if there was a rupture of a cavernoma but there is no contraindication to starting antiplatelets/anticoagulation in her. The patient agreed to start an anticoagulant, which was decided to be started two weeks after her stroke due to the size of the PCA infarct. A repeat CT head was done at that time, which showed stable infarct territory. Eliquis 5 mg twice a day was initiated based on insurance coverage cost, and in the neurologic clinic follow-up, the patient was tolerating the medication with the resolution of her dizziness and visual symptoms from the PCA infarct. She later followed up with cardiology in the outpatient setting who recommended the left atrial appendage occlusion (LAAO) procedure as treatment for her atrial fibrillation.

## Discussion

Atrial fibrillation significantly increases the risk of stroke, with the risk being five times higher in individuals with the condition compared to the general population [4]. The estimated national prevalence of diagnosed atrial fibrillation is at least 10.55 million (95% CI 10.48-10.62 million), comprising 4.48% (95% CI 4.47%-4.49%) of the adult population [5]. Over time, atrial fibrillation can progress from paroxysmal (intermittent) to persistent or permanent forms, particularly in cases where it remains untreated. This progression can lead to a greater risk of hospitalizations and a higher risk for complications like stroke and reduced quality of life [6]. For the initiation of oral anticoagulation in non-valvular atrial fibrillation, the CHA2DS2-VASc (congestive heart failure, hypertension, age ≥ 75 (doubled), diabetes, stroke (doubled), vascular disease, age 65-74, and sex category (female)) score is used to assess a patient’s risk of stroke and systemic thromboembolism. A recent analysis of the net clinical benefit of a vitamin K antagonist in patients assessed for stroke risk by CHA2DS2-VASc and HAS-BLED (hypertension, abnormal renal/liver function, stroke, bleeding history or predisposition, labile international normalized ratio (INR), elderly, drugs/alcohol concomitantly) suggested a net positive benefit (measured as the balance between ischemic stroke and intracranial hemorrhage) in patients with a CHA2DS2-VASc score greater than or equal to 2 and even higher benefit in patients with a HAS-BLED score of 3 or greater. A negative net clinical benefit was only seen in patients with a CHA2DS2-VASc score of 0, reflecting their truly low-risk status for stroke. Thus, in order to obtain a net positive benefit from antithrombotic therapy, careful consideration must be made for patient populations at risk for serious bleeding complications. In our patient, she met the need for anticoagulation for secondary stroke prevention. Given the patient’s known history of CMM, the initial worry was how this would increase her risk of hemorrhage or rupture.

Coordination of care across several specialties is often needed to optimize anticoagulation management in patients with CCMs. Therefore, decision-making often includes the expertise of cardiology, neurology, neurosurgery, and radiology. In the presented clinical scenario, for example, the neurology team first provided diagnostic clarity on the type of stroke the patient suffered (i.e., cardioembolic secondary to atrial fibrillation). Cardiology was consulted to help determine the risks/benefits of anticoagulation as part of long-term atrial fibrillation management with additional considerations for interventional procedures (e.g., catheter ablation, pacemaker placement, and left atrial appendage closure). While it is very common for the neurology-cardiology relationship to revolve around stroke prevention vs. bleeding risks, it does not often involve the variable of CCM hemorrhage. Therefore, it was most within neurology's expertise to provide insight into the potential risks of hemorrhage from these vascular lesions if anticoagulation was initiated. Radiology influenced decision-making by identifying risk factors for CCM bleeding such as size, location, and prior hemorrhage. Lastly, neurosurgical consultation was obtained to determine if the operative removal of the CCMs was warranted. Collaborating across these specialties ensures that therapies are tailored to the individual patient and that the most appropriate treatment option is selected.

While the majority of CCMs remain clinically silent, these vascular anomalies are associated with a 2%-6% chance of rupture per year [1]. The factors associated with bleeding risk remain unclear, thus posing a challenge to clinicians on how to medically or surgically manage these patients. A systematic review of patient data taken from cohort studies following the clinical course of untreated CCM identified that significant predictors for risk of hemorrhage within five years of diagnosis include the location of the lesion, specifically the brainstem, and the mode of presentation with intracerebral hemorrhage (ICH) or focal neurological deficits [2]. One retrospective observation study determined that the only independent predictor of bleeding was median CCM volume (adjusted OR 3.11 (95% CI 1.09-8.86)) [1]. Meanwhile, other modifiable risk factors such as hypertension, age, and sex may play a role but did not reach statistical significance for prognostic value and had much lower sensitivity.

An important factor to consider for our case is how medications play a role in the evolution of CCM. Current data in the literature seem to suggest that antithrombotic agents, which include antiplatelet and anticoagulant medications, are not associated with an increased bleeding risk [1,3,7]. A systematic review and meta-analysis conducted by Musmar et al. found that patients with CCM on antithrombotic therapy had a lower risk of symptomatic presentation with ICH (OR 0.56 (95% CI 0.45-0.7); p < 0.0001), and the use of antithrombotic therapy was associated with a decreased risk of progression to intracranial hemorrhage from CCM during follow-up (OR 0.21 (95% CI 0.13-0.35); p < 0.0001) [3]. Recent studies suggest that beta-blockers such as propranolol may confer therapeutic benefits by causing the regression of CCM to prevent recurrent bleeding [8]. Similarly, another study demonstrated further evidence that antithrombotic therapy alone or in combination with statins in patients with CCMs has a lower risk of hemorrhage or focal neurological deficits compared to patients not on any treatment [7]. The significance of all these findings suggests that antithrombotic agents may be associated with a lower risk of intracranial hemorrhage in patients with CCMs, which challenges the conventional recommendations that anticoagulation was contraindicated for people with CCM. Furthermore, the overall risk of death and mortality conferred by bleeding in CCM is relatively low, estimated at 2.2%, but progressive neurologic deficits can accumulate and significantly impact a patient's quality of life [9].

LAAO is also now emerging as an alternative to anticoagulation in those with atrial fibrillation [10]. Thus far, clinical trials have shown non-inferiority of LAAO when compared to oral anticoagulants. This makes LAAO an important clinical management option in those with contraindications to oral anticoagulants and, thus, should involve discussions with cardiology.

In conclusion for our patient, anticoagulation was deemed safe for secondary stroke prevention. Due to the size of her infarct territory, a delay in the initiation of anticoagulation was done. Eventually, cardiology plans to do a LAAO procedure to eventually take her off anticoagulation.

## Conclusions

The use of anticoagulation for secondary stroke prevention in patients with CCM remains a complex and challenging clinical scenario due to the fear of hemorrhagic complications. Surprisingly, prior meta-analysis found that the CCM population has an overall decreased risk of symptomatic intracranial hemorrhage while on antithrombotic therapy. Independent bleeding risks to take into account before adding such medications include patient age, infratentorial location, CCM size, and the association with a developmental venous anomaly. As such, there is no absolute contraindication to the initiation of anticoagulation for secondary stroke prevention in patients with CCM; however, this decision must be individualized with careful analysis of the benefits vs. hemorrhage risk. Multidisciplinary consultation involving input from neurology, neurosurgery, cardiology, and radiology is also often crucial to optimizing patient outcomes in this setting. This case demonstrates the considerations, safety, and efficacy of initiating anticoagulation for a middle-aged female patient with multiple known intracranial CCMs who was found to have an acute right P1 PCA occlusion likely due to cardioembolism in the setting of atrial fibrillation.
